# The effect of non-uniformity fractionation for tumor metastatic abilities

**DOI:** 10.1093/jrr/rrt185

**Published:** 2014-03

**Authors:** Yoshitaka Matsumoto, Kei Yamashita, Huizi Li, Yoshiya Furusawa

**Affiliations:** 1 Research Center for Charged Particle Therapy, National Institute of Radiological Sciences, 4-9-1 Anagawa, Inage-ku, Chiba 263-8555, Japan; 2 Graduate School of Medicine, Chiba University, Chiba, Japan

**Keywords:** non-uniformity fractionation, metastasis, carbon-ion beam, low-dose

## Abstract

Purpose/background: In recent years, the local control of tumor after radiotherapy has shown a remarkable improvement due to the widespread of the particle beam therapy, IMRT etc.. However, the survival rate of the patients is not satisfied because of the recurrence or distant metastasis. There is an urgent need to search for therapies to control the metastasis. Hyperfractionation is the mainstream of the recent radiotherapy, and the fraction size is getting smaller. Our previous study, however, showed the migration and invasion abilities of tumor cells were enhanced by low-LET radiations at the low-dose region. Small fraction size in hyperfractionation might promote the metastasis. The aim of this study is to examine the effect of non-uniformity fractionation for cell survival and metastatic potential.

Materials and methods: LM8 cells having highly metastatic potential was used and cells were irradiated with two fractionated exposure (24 h interval) in this study. X-rays and carbon-ion beams were used as low- and high-LET irradiation, respectively. First non-uniformity fractionation method: cells were irradiated with the same total dose but different dose allocation (e.g. total 2 Gy, first dose is 0.5 or 1.5 Gy and second dose is 1.5 or 0.5 Gy). Second method: cells were first irradiated with C-ions and then, with X-rays.

Results/discussion: The fractionation with large initial dose decreased the cell survival and migration ability compared with small initial dose even the same total dose (Fig. 1). Additionally, the fractionation with high-LET initial radiation decreased the cell survival and migration ability compared with low-LET initial irradiation, and this effect was significant at low-dose region, especially (Fig. 2).

It is suggested that non-uniformity fractionation using the high dose and/or high-LET beams as the initial irradiation suppressed efficiently the cell survival and metastatic potential compared with uniformity fractionation and the non-uniformity fractionation may effective from viewpoint of the suppression of tumor metastasis.

Conclusion: The non-uniformity dose/LET fractionation would be effective to suppress not only the tumor cell survival and tumor metastatic abilities.

**Fig. 1.**
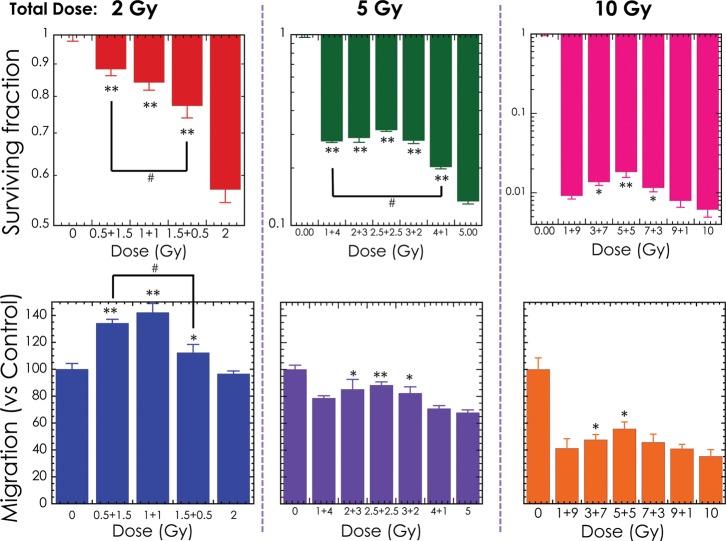
The surviving fraction and migration of LM8 cells after non-uniformity dose X-rays irradiation. *0.01 < *P* < 0.05, **P < 0.01, compared with one fractionation samples. ^#^0.01 < *P* < 0.05, compared between the same total dose samples.

**Fig. 2.**
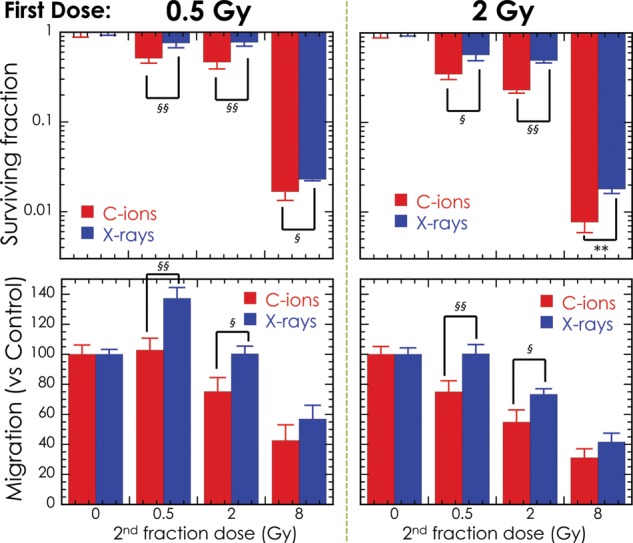
The surviving fraction and migration of LM8 cells after non-uniformity LET irradiation using X-rays and carbon-ion beams. ^§^0.01 < *P* < 0.05, ^§§^*P* < 0.01, compared between X-rays and carbon-ion beams samples.

The surviving fraction and migration of LM8 cells after non-uniformity dose X-rays irradiation. *0.01 < *P* < 0.05, **P < 0.01, compared with one fractionation samples. ^#^0.01 < *P* < 0.05, compared between the same total dose samples.

The surviving fraction and migration of LM8 cells after non-uniformity LET irradiation using X-rays and carbon-ion beams. ^§^0.01 < *P* < 0.05, ^§§^*P* < 0.01, compared between X-rays and carbon-ion beams samples.

